# Supplementary material for: Suicide after leaving the UK Armed Forces 1996–2018: A cohort study
Source: PLoS Med. 2023 Aug 8;20(8):e1004273. doi: 10.1371/journal.pmed.1004273 (PMC10409259; doi:10.1371/journal.pmed.1004273)
Supplement: S1 STROBE Checklist — (DOCX) [file pmed.1004273.s001.docx]

STROBE Statement—Checklist of items that should be included in reports of ***cohort studies***

|  | Item No | Recommendation | Page No |
| --- | --- | --- | --- |
| **Title and abstract** | | | |
|  | 1 | (*a*) Indicate the study’s design with a commonly used term in the title or the abstract | Title & “Methods and findings” section of the Abstract. |
|  |  | (*b*) Provide in the abstract an informative and balanced summary of what was done and what was found | “Methods and findings” and “Conclusion” sections of the Abstract. |
| Introduction | | | |
| Background/rationale | 2 | Explain the scientific background and rationale for the investigation being reported | “Introduction” section of the main manuscript. |
| Objectives | 3 | State specific objectives, including any prespecified hypotheses | “Background” section of the Abstract. Final paragraph of the “Introduction” section of the main manuscript. |
| Methods | | | |
| Study design | 4 | Present key elements of study design early in the paper | “Methods and findings” section of the Abstract. “Study design” subsection (paragraph 1) of the “Methods” section of the main manuscript. |
| Setting | 5 | Describe the setting, locations, and relevant dates, including periods of recruitment, exposure, follow-up, and data collection | “Methods and findings” section of the Abstract. “Study setting and individuals” subsection (paragraph 2) of the “Methods” section of the main manuscript. |
| Participants | 6 | (*a*) Give the eligibility criteria, and the sources and methods of selection of participants. Describe methods of follow-up | “Study setting and individuals” subsection (paragraph 2) of the “Methods” section of the main manuscript. |
|  |  | (*b*) For matched studies, give matching criteria and number of exposed and unexposed | “Statistical analysis” subsection (paragraph 8) of the “Methods” section of the main manuscript. |
| Variables | 7 | Clearly define all outcomes, exposures, predictors, potential confounders, and effect modifiers. Give diagnostic criteria, if applicable | “Method” section of the main manuscript |
| Data sources/ measurement | 8* | For each variable of interest, give sources of data and details of methods of assessment (measurement). Describe comparability of assessment methods if there is more than one group | “Method” section of the main manuscript |
| Bias | 9 | Describe any efforts to address potential sources of bias | “Strengths and limitations” subsection (paragraph 22) of the “Discussion” section of the main manuscript |
| Study size | 10 | Explain how the study size was arrived at | N/A: national data |
| Quantitative variables | 11 | Explain how quantitative variables were handled in the analyses. If applicable, describe which groupings were chosen and why | “Statistical analysis” subsection (paragraph 8) of the “Methods” section of the main manuscript. |
| Statistical methods | 12 | (*a*) Describe all statistical methods, including those used to control for confounding | “Statistical analysis” subsection (paragraph 8) of the “Methods” section of the main manuscript. |
|  |  | (*b*) Describe any methods used to examine subgroups and interactions | “Statistical analysis” subsection (paragraph 8) of the “Methods” section of the main manuscript. |
|  |  | (*c*) Explain how missing data were addressed | “Statistical analysis” subsection (paragraph 8) of the “Methods” section of the main manuscript. |
|  |  | (*d*) If applicable, explain how loss to follow-up was addressed | N/A |
|  |  | (*e*) Describe any sensitivity analyses | N/A |
| **Results** | | | |
| Participants | 13* | (a) Report numbers of individuals at each stage of study—eg numbers potentially eligible, examined for eligibility, confirmed eligible, included in the study, completing follow-up, and analysed | “Database linkage” subsection (paragraph 7) of the “Methods” section of the main manuscript. “Results” section of the main manuscript (paragraphs 10, 11, and 17). |
|  |  | (b) Give reasons for non-participation at each stage | N/A |
|  |  | (c) Consider use of a flow diagram | N/A |
| Descriptive data | 14* | (a) Give characteristics of study participants (eg demographic, clinical, social) and information on exposures and potential confounders | “Results” section and Tables 2 & 3 of the main manuscript |
|  |  | (b) Indicate number of participants with missing data for each variable of interest | The valid N is given in the “Results” section of the main manuscript and in Tables 1 to 4 |
|  |  | (c) Summarise follow-up time (eg, average and total amount) | N/A |
| Outcome data | 15* | Report numbers of outcome events or summary measures over time | N given in Results (pages 11-14) and Tables (pages 27, 29-32) |
| Main results | 16 | (*a*) Give unadjusted estimates and, if applicable, confounder-adjusted estimates and their precision (eg, 95% confidence interval). Make clear which confounders were adjusted for and why they were included | “Results” section and Tables 1-4 of the main manuscript. |
|  |  | (*b*) Report category boundaries when continuous variables were categorized | N/A |
|  |  | (*c*) If relevant, consider translating estimates of relative risk into absolute risk for a meaningful time period | N/A |
| Other analyses | 17 | Report other analyses done—eg analyses of subgroups and interactions, and sensitivity analyses | “Statistical analysis” subsection (paragraph 8) of the “Methods” section of the main manuscript. “Results” section of the main manuscript. Tables 1-4, Figure 1. |
| **Discussion** | | | |
| Key results | 18 | Summarise key results with reference to study objectives | “Main findings” subsection (paragraphs 20 and 21) of the “Discussion” section of the main manuscript. |
| Limitations | 19 | Discuss limitations of the study, taking into account sources of potential bias or imprecision. Discuss both direction and magnitude of any potential bias | “Strengths and limitations” subsection (paragraph 22) of the “Discussion” section of the main manuscript |
| Interpretation | 20 | Give a cautious overall interpretation of results considering objectives, limitations, multiplicity of analyses, results from similar studies, and other relevant evidence | “Interpretation of findings” subsection (paragraph 23) of the “Discussion” section of the main manuscript |
| Generalisability | 21 | Discuss the generalisability (external validity) of the study results | “Discussion” section of the main manuscript |
| **Other information** | | | |
| Funding | 22 | Give the source of funding and the role of the funders for the present study and, if applicable, for the original study on which the present article is based | Available in the manuscript metadata |

*Give information separately for exposed and unexposed groups.

**Note:** An Explanation and Elaboration article discusses each checklist item and gives methodological background and published examples of transparent reporting. The STROBE checklist is best used in conjunction with this article (freely available on the Web sites of PLoS Medicine at http://www.plosmedicine.org/, Annals of Internal Medicine at http://www.annals.org/, and Epidemiology at http://www.epidem.com/). Information on the STROBE Initiative is available at http://www.strobe-statement.org.
