# Supplementary figures and images for: Suicide after leaving the UK Armed Forces 1996–2018: A cohort study
Source: PLoS Med. 2023 Aug 8;20(8):e1004273. doi: 10.1371/journal.pmed.1004273 (PMC10409259; doi:10.1371/journal.pmed.1004273)

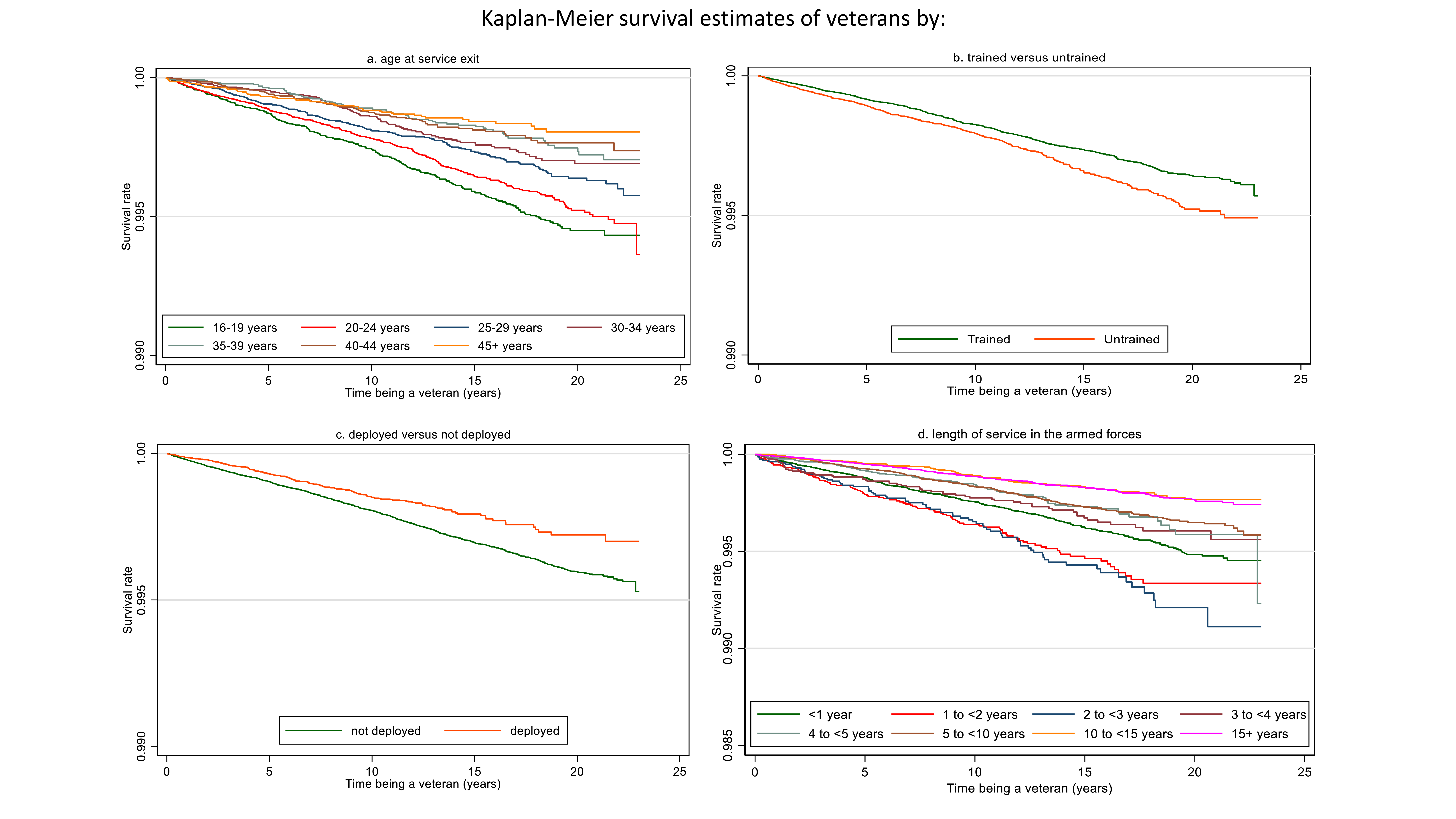

Supplement: S1 Fig — Kaplan–Meier survival estimates of veterans by: (a) age at service exit; (b) trained versus untrained; (c) deployed versus not deployed; (d) length of service in the UKAF. (TIFF) [file pmed.1004273.s002.tiff]
