# Supplementary material for: Suicide after leaving the UK Armed Forces 1996–2018: A cohort study
Source: PLoS Med. 2023 Aug 8;20(8):e1004273. doi: 10.1371/journal.pmed.1004273 (PMC10409259; doi:10.1371/journal.pmed.1004273)
Supplement: Attachment — Submitted filename: rodway.pdf [file pmed.1004273.s003.pdf]

This paper uses existing databases to assess the characteristics of suicides in UK veterans.

I was asked for a statistical report and I interpret that to include all aspects of the design and conduct of the study.

Points of detail

Page 7 What is the difference between a death registered as suicide and one registered as intentional self–harm? Is this a distinction which will be understood by an international readership?

Page 8 I think this is one of the rare cases where results (the actual numbers linked) do belong in the methods not the results.

Page 9 I assume 'Ethnics' is a typo?

Page 12 Some of the detailed comment here about age relies on eyeballing the data. If the reported differences are important then I think they need backing up with some more formal approach.

Page 13 Having said that female data is not to be provided by age due to small numbers the authors then discuss it. I think it is a shame not to accord women the same treatment as men so I would suggest presenting it. The confidence intervals will be wide of course. International researchers might want to meta–analyse female data so putting it out there would help them.

Page 13 The authors state

> There was possible evidence of an increase in risk between years 8 and 14 after discharge, which may have reflected the underlying age of the veteran cohort.

Really? To my eyes that is not what Figure 1 shows. Year 14 is high but that is one year out of 16. Page 20 also mentions this.

Page 18, Table 3 This seems to have some entries in bold. If you are going to do that then do it consistently across all the tables but I would suggest it is not a good idea. Leave it to the readers to decide what to look at. I applaud the attempt to give the exact p–values but it does make the table rather ugly. More decimal places might help, there is room.

Page 18, Table 3 I do not understand the footnote about χ^2. Are these not supposed to be from a conditional logistic regression? Are these

really unadjusted for covariates?

Page 20 If suicide methods are sex dependent then does it make sense to compare general populations? I agree this may mean we can only see male results but they would be less contaminated.

Supplement I suspect RECORD (Benchimol et al., 2015) may be the more appropriate guideline. I did not notive any obvious omissions.

Points of more substance

Age categories

Different analyses use different categorisations. I can see why that might be but an explicit statement might be needed. Some of the tables provide that.

Plotting with a superimposed curve (perhaps loess) might help enlighten us about the age relationships. Table 1 is an example. The plot could then use age in years which is less arbitrary than categorised age.

Models

Presumably if you are neither an officer nor an NCO you are a private soldier or equivalent. There seem to be a lot of them (Page 15, Table 2). Coupled with the high proportion discharged as untrained makes me suspect that many of these may have been early discharges. The length of service data seems to confirm that. Is that true? As a UK taxpayer I would hope our armed forces were not quite so untrained.

I think that:

1. The fact that these are unadjusted models needs specifying in the text not just in the caption.
2. The discussion needs to take account of the fact that, if I am correct, the correlated covariates are saying the same thing three times not three separate things.
3. If they are correlated the multivariable model could be fitted and the adjusted coefficients presented alongside the unadjusted ones which may prove enlightening.

It would also be better to provide an overall test for each covariate with more than two levels not just test levels against the reference.

Summary

Points of detail and some queries about the models.

Michael Dewey

References

E I Benchimol, L Smeeth, A Guttmann, K Harron, D Moher, I Petersen, H Sørensen, E von Elm, S Langan, and RECORD working committee. The REporting of studies Conducted using Observational Routinely–collected health Data(RECORD) Statement. *PLOS Medicine*, 12(10), 2015.
