# Supplementary material for: Suicide after leaving the UK Armed Forces 1996–2018: A cohort study
Source: PLoS Med. 2023 Aug 8;20(8):e1004273. doi: 10.1371/journal.pmed.1004273 (PMC10409259; doi:10.1371/journal.pmed.1004273)
Supplement: Attachment — Submitted filename: Response to reviewers comments.docx [file pmed.1004273.s004.docx]

**Manuscript ID: PMEDICINE-D-22-04050R1**

**Manuscript title: Suicide after leaving the UK Armed Forces 1996-2018: a cohort study**

**Responses to reviewers’ comments**

**Editorial requests**:

1. We trust you will include additional details of the calculations, and if needed additional analyses, to respond to referee 1's comments.

**Response**: We can confirm that additional information on our analysis has been added to the statistical analysis subsection of the methods and additional analyses, where necessary, have been performed in response to all reviewer comments.

2. Please add a new final sentence to the 'Methods and findings' subsection of the abstract which should begin "Study limitations include ..." or similar and should quote 2-3 of the study's main limitations.

**Response**: As requested, study limitations have been added to the end of the “methods and findings” subsection of the abstract on page 2, lines 34-39.

3. At line 36 (abstract), please consider adding "Study limitations include ..." or similar.

**Response**: Please see our response to comment 2 above.

4. Please add an accessible 'Author summary' section after the Abstract, in non-identical prose. You may find it helpful to consult one or two recent research articles in PLOS Medicine to get a sense of the preferred style.

**Response**: An “author summary” section has now been added to pages 3-4, lines 47-81, of the manuscript.

5. Please trim the final paragraph of the Introduction (main text) to remove elements of discussion. Stating the aim of the study will suffice here.

**Response**: The final paragraph of the introduction (main text) has been trimmed, as requested.

6. Please remove information on study funding from the end of the main text. In the event of publication, this information will appear in the article metadata via entries in the submission form.

**Response**: The information on study funding has been removed from the main text, as requested.

7. In the reference list, please convert all italics to plain text.

**Response**: All italics have been converted to plain text in the reference list.

8. Noting references 21 and 22, please list 6 author names consistently followed, where appropriate, with "et al.".

**Response**: References 22 and 23 have been corrected, as has reference 40.

9. Thank you for including the completed STROBE checklist. Please rename the attachment 'S1_STROBE_Checklist" or similar and refer to it in the Methods section (Main text).

**Response**: We have renamed the attachment as ‘S1_STROBE_Checklist’, as requested, and have referred to it in the Methods section (page 8, lines 150-152).

10. In the checklist, please refer to items by section (e.g., 'Methods') and paragraph number, not by page numbers.

**Response**: Items in the STROBE checklist are now referred to by section and paragraph number, not page numbers, as requested.

**Reviewer #1:**

Thank you for the opportunity to review the revised version of this extremely important paper, a data linkage study of veteran suicides over the period 1 Jan 1996-31 Dec 2018. It is likely that this paper will inform both Government and Defence policy on suicide prevention, and therefore it is crucial that the findings are both valid and robust.

The authors had access to two large national databases, one recording Service leavers and one recording suicides and open verdicts nationally and performed a data linkage to identify probable suicides among people with previous military service.

1. A total of 1,086 suicides (0.2%) were identified among the 458,048 veterans who entered the database between 1 January 1996 and 31 December 2018. This is stated to cover a 22-year period (lines 21 and 131); I calculate it to cover 23 years.

**Response:** Thank you for highlighting this error. It is indeed a 23-year period and we have corrected this in the abstract (page 2, line 21).

2. I have considerable concerns with regard to the statistical analysis which is presented in the Results, particularly in lines 238-249. Table 1, showing a steady decline in "crude rate" from the youngest at death (16-19 years of age) to the oldest (45-54 years of age) is at marked variance with the nationally-reported age-specific rates for suicide, which show a near-normal distribution centred around middle age. It is difficult to understand how it could be plausible that a short period of military service (the median age at discharge for those who later died was 23 years) would so radically change the overall pattern of age at suicide. National data are based on annual prevalence rates per 100,000 population, but this becomes problematic when looking at veterans as the numbers in each age category are relatively small, eg a mean of less than 2 cases per year for the category aged 16-19 years at death as reported in Table 1. The authors have therefore used a rate of "per 100,000 person-years at risk". However they have not described how this was derived in the Methods, and I presume that it represents elapsed time between discharge from the military and death. The flaw in this method is that the longer the time between discharge and death (ie generally, the older the individual is at death), the more person-years they have been "at risk", and therefore the larger the denominator. This inevitably results in the declining "crude rate" seen in the third column of Table 1, but it is an artefact of the methodology, not a reduction in risk in older veterans. This has significant implications for policy.

**Response**: Thank you very much for these thoughtful comments. We wholeheartedly agree that our findings need to be valid and robust and particularly appreciated the reminder about policy implications. We are confident that are results are not merely an artefact of our analytic strategy:

- We would not expect the distribution of the suicide rate in this population to be the same as for the general population because the veterans in this study are not a random sample of the UK population as a whole. They have been selected on the basis of past military service, are overwhelmingly male, and younger than the general population.
- The reviewer mentions the plausibility of such a short length of service shifting the distribution of suicide. This assumes a causal effect of service on suicide - we think the observed findings are much more likely to be a selection effect (that is, they reflect the underlying characteristics of the at-risk veteran cohort).
- The reviewer is correct in their suggestion that the number of individual deaths by age category in individual years is small. We used a standard and widely accepted epidemiological approach to the calculation of incident rates in dynamic cohort data, i.e. a person-years at risk denominator (Checkoway et al (2004) provide a useful summary).
- We agree with the reviewer that we should have provided further details of how the denominators were derived. We have now included these details in our analysis section. To reassure the reviewer, the person years at risk does not represent a cumulative total for each individual. Instead, each individual contributes person years at risk to successive age categories as they get older. This means the person years denominators used to calculate the crude rates in Table 1 take into account that an individual’s age moves between age categories as the study progresses. For example, an individual who left the UKAF on their 26^th^ birthday and died aged 46 years and 6 months would contribute 4 person-years at risk to the 25-29-year age band, 5 person-years at risk to each of the 30-34-, 35-39-, and 40-44-year age bands, and 1.5 person-years at risk to the 45-49-year age band.
- We structured the data to be able to carry out the analysis in this way and used the STATA command STPTIME to calculate age-specific rates and SMR so the person-time at risk moved along the age bands. Further detail regarding this analysis has been added to the statistical analysis subsection of the methods on page 11, lines 230-233.
- Furthermore, the declining crude rates in older age groups are not merely a function of the denominators – the number of suicide deaths in veterans in Table 1 show a marked decline too.
- We therefore do not believe that our findings on young veterans are an artefact of the methodology. The findings are consistent with our earlier study (Kapur et al, 2009) and with recent findings from a major US report of suicide in veterans (US Department of Veteran Affairs, 2022).
- However, we do appreciate the challenges and sensitivities around translating this research into policy implications. For example, the recent Scottish study of military veterans which was mentioned (Bergman et al, 2022) identified a different at-risk group (men and women in middle-age), which is likely to reflect differences in study design and setting, inclusion criteria, and time period. We have added some text to the discussion to acknowledge this (see page 24, lines 496-503).
- If we put the absolute rates of suicide in our study to one side for the moment, in terms of numbers 16-24-year-olds formed 18% of the total veteran population who died by suicide; older age groups (aged 40 to 54 years) made up 26% (even though their actual rates are lower). It’s therefore clear that veterans across the age range, are also an important part of the prevention challenge. We have added text to our abstract, lay summary, interpretation and implications sections of the discussion to reflect this.

3. These observations are also relevant to Table 4.

**Response**: Please see our response to comment 2 above. Please also note, Table 4 has been updated to include males serving in the UKAF who died in-service in 1996 and veterans’ who died in 1996 to be in line with the rates presented in Table 1.

4. The fourth column of Table 1 presents age-specific mortality ratios, using the general population as the reference population. The rates for the veterans are presumably those presented in column 3; the derivation of the corresponding rates for the population is not described but presumably comes from published national data. Are the population rates averaged over 23 years as for the veterans, or has a single year's rates been used and if so, which year?

**Response**: Thank you for your comment. Yes, the rates for veterans are those presented in column 3 of Table 1 and have now been labelled as such (also in Table 4). The corresponding rates for the general population were calculated using the number of suicides from the NCISH general population database over the study period as the numerator and annual published mid-year population estimates from ONS as the denominator. As the reviewer suggests, this means we are essentially comparing average rates of suicide in veterans and the general population across the whole study period. We have now described how rates were calculated in the statistical analysis subsection of the methods on page 11, lines 238 to 241, including a reference to the published ONS data. We have also added this reference to Table 1. We have made additional clarifications to the tables in the manuscript.

5. Furthermore, and importantly, the validity of calculating a ratio based on a comparison between a crude rate derived from person-years since leaving service in one population, and an annual incidence in another population, must be in question.

**Response**: The comparison of incidence rate data based on person years at risk with general population rates is a fairly standard epidemiological approach when calculating SMRs. Again, the article by Checkoway et al (2004) provides further details.

6. Similar concerns apply to Figure 1, which also presents rate per 100,000 person-years at risk, by years since discharge. The number of person-years at risk (and hence the denominator) inevitably rises with years since discharge, so the methodology may have resulted in this important figure concealing the true picture. Again, there is an inconsistency; the median interval between discharge and death was 9 years (from the second paragraph of the Results) but that is in no way reflected in Figure 1.

**Response**: Again, we can reassure the reviewer that the persons years at risk do not simply accumulate with years since discharge - an individual will contribute a single year at risk for every time interval in Figure 1. The confusion may have arisen because we did not make it clear that the values on the X-axis were interval data. The new Figure 2 (it has been renumbered because we have included an additional figure as requested by reviewer 2) has now been relabelled to clarify this. The rates presented in Figure 2 are for each interval per 100,000 person years at risk. This means that a person discharged at the start of 2013 who died by suicide at the end of 2015 would contribute 1 person year at risk to the 0-1 years since discharge interval, 1 person year at risk to the 1-2 years discharge interval. We would not expect the median interval between discharge and death of 9 years to be reflected in Figure 2 since it plots rates rather than frequencies.

7. Overall, it is suggested that survival analysis (Cox proportional hazard analysis) would be a better methodology for assessing risk of suicide, using age as the time-dependent covariate instead of time since leaving service, and using age at suicide as the failure time. A comparison group of non-veterans could be selected, which would allow valid comparison with the general population. Calculation of hazard ratios for a wide range of subgroups of interest would be facilitated, particularly for those putative risk factors where a general population comparator was appropriate.

**Response**: We thank the reviewer for this suggestion and agree this is an elegant methodology. However, it necessities a general population control group on who equivalent data are available. This would represent a huge undertaking and a different study to the one described in this manuscript. In the absence of comprehensive national databases, it would not have been feasible for us to do this. We do acknowledge, however, that previous research has used this methodology (for example, the Scottish veterans’ study) and have added this as a potential weakness of our investigation. Of course, an advantage of the current methodology is that it is a whole population approach with a large sample size. Further text has been added to the strengths and limitations subsection of the discussion on page 22, lines 450 to 453.

8. Lines 19 and 20 of the Abstract state that there have been no recent UK studies investigating veteran suicide. It would be more accurate to say "no recent UK-wide studies" since Reference 11 is a study of Scottish veterans published in 2022.

**Response**: Thank you for your suggestion, line 20 of the abstract has been amended to read “…and no recent UK-wide studies”.

9. Line 88 refers to the 2011 Armed Forces Covenant. For completeness, and to bring the Government perspective on caring for veterans up to date, the 2018 Veterans' Strategy and 2022 Veterans' Strategy Action Plan should also be mentioned here.

**Response**: Thank you for your comment. A reference to the Veterans’ Strategy Action Plan has been added to the introduction. Pages 6-7, lines 133-135, now reads “Most recently, the Veterans’ Strategy Action Plan has detailed the steps the UK Government will take to better support the veteran community by 2028”.

10. The authors should consider noting (either in line 94 of the Introduction and/or under Limitations) the marked geographical variation in suicide rates in England and Wales as reported in Reference 34, ranging from 6.6 per 100,000 in London to 14.1 per 100,000 in the North-East, and should discuss the implications for interpretation of their results.

**Response**: As suggested, geographical variations in suicide rates are now referred to in the strengths and limitations subsection of the discussion on page 23, lines 474-483:

“In our analysis we calculated SMRs using the aggregated number of suicide deaths from the NCISH general population database and UK population estimates [42]. This approach, however, did not take into account levels of deprivation or consider geographical variations in suicide rates [37]. It is difficult to be certain whether or how this may have impacted our findings – basing calculations on general population rates in the North-East of England (which has a high general population suicide rate) would have led to lower SMRs for the veterans’ population than using London rates (which have lower general population suicide rates). One approach might be to carry out analysis stratified by geographical area. We will be exploring spatial distribution of veterans’ suicide deaths in future work, which will contextualise our findings on whether suicide risk in younger veterans is associated with the areas in which they live following discharge from the UKAF.”

11. Line 117: Add at end of sentence "in accordance with the UK definition of a veteran".

**Response**: As suggested, we have added “in accordance with the UK definition of a veteran” to line 159 of the subsection ‘study setting and individuals’, page 8. We have also cited the Armed Forces Covenant here, which provides this definition of a veteran.

12. Line 130: The comma between staffing and surveys should be removed.

**Response**: Thank you for highlighting this error – the comma (on what is now line 173) has been removed.

13. Line 189: The description of the Cox proportional hazard analysis should specify the time-dependent covariate and failure time.

**Response**: Thank you for your comment. As suggested, the description of the Cox proportional hazard model now specifies the time-dependent covariate and failure time:

“To investigate the risk factors that were most associated with suicide by veterans after discharge we used Cox proportional hazard models with age at death as the failure time and the end of the study period (31 December 2018) as the censor time” (see page 11, lines 241 to 243).

14. Line 240: The words "If anything" should be replaced with "Overall".

**Response**: The wording has been amended to “overall” at line 297 of the manuscript, as suggested.

15. Line 392: It would be worth clarifying " . . veterans who had served solely as reserves . ." as a number of people go on from Regular service to join the Reserves and presumably they would not have been excluded from consideration as Regulars.

**Response**: Thank you for your comment. The reviewer is correct that individuals who moved from the Regulars to join the Reserves were *not* excluded from consideration as Regulars. Lines 485 and 488 on pages 23 and 24 have therefore been amended to “veterans who had served *only* as reserves…”, as suggested.

16. Under Limitations, the lack of adjustment for socio-economic status should be mentioned, as should the lack of adjustment for geographical variations in background population suicide rate, as noted above. The relatively short length of the study, limiting the number of veteran suicides able to be included in the analysis, should also be mentioned here, albeit it was constrained by the availability of relevant data as noted in the Methods.

**Response**: Under the strengths and limitations section (pages 22-24, lines 439-483) we have added lack of adjustment for socio-economic status or geographical variations in suicide rates, and the relatively short length of the study as methodological shortcomings of our study, as suggested by the reviewer. However, as mentioned above (see our response to your comment #10), we do plan to examine the spatial distribution of veterans’ suicide deaths in a further paper.

**Reviewer #2:**

This paper uses existing databases to assess the characteristics of suicides in UK veterans. I was asked for a statistical report and I interpret that to include all aspects of the design and conduct of the study.

1. Page 7 What is the difference between a death registered as suicide and one registered as intentional self–harm? Is this a distinction which will be understood by an international readership?

**Response**: Thank you for your comment. To clarify, a conclusion of suicide is given at coroner inquest, whereas “intentional self-harm” refers to ICD-10 coding of deaths. The methods section on pages 9-10, lines 192-196, has now been amended:

“Deaths assigned International Classification of Diseases, Tenth Revision (ICD-10) codes X60-X84 (intentional self-harm) or ICD-10 codes Y10-Y34, excluding Y33.9, Y87.0, and Y87.2 (events of undetermined intent) were included in the study sample, as is standard for suicide research in the UK (since the majority of these undetermined deaths are suicides) [41]. These deaths will collectively be referred to as suicide deaths throughout this paper.”

2. Page 8 I think this is one of the rare cases where results (the actual numbers linked) do belong in the methods not the results.

**Response**: Thank you for your comment. We are pleased that you agree that the actual numbers linked between the two databases are better presented in the methods not the results, as we have done.

3. Page 9 I assume ‘Ethnics’ is a typo?

**Response**: Thank you for highlighting this. The typo has been corrected.

4. Page 12 Some of the detailed comment here about age relies on eyeballing the data. If the reported differences are important then I think they need backing up with some more formal approach.

**Response**: Thank you for your comment, to improve clarity the sentence [on what is now page 14] has been amended to “Overall, the suicide rate appeared to fall with age from 28.0 per 100,000 person-years at ages 16-19 to 8.8 per 100,000 person-years at ages 55-59 (Table 1, Figure 1)” (lines 304-305). Following your comment below (#13) a Lowess curve has also been added to the manuscript here.

5. Page 13 Having said that female data is not to be provided by age due to small numbers the authors then discuss it. I think it is a shame not to accord women the same treatment as men so I would suggest presenting it. The confidence intervals will be wide of course. International researchers might want to meta–analyse female data so putting it out there would help them.

**Response**: The reviewer makes a fair comment about the exclusion of female data from the manuscript. However, female veterans made up a very small proportion of those who died by suicide (n=40, 4%) and cell counts were low (less than 5) in some age categories – too low for us to be able to report them according to Ministry of Defence guidelines on disclosure control to protect confidentiality (MoD 2022 Joint Service Publication 200: Statistics section 4.12 Disclosure Control). We have therefore not included these data but have added a statement about small numbers and disclosure control to the statistical analysis subsection in the methods (page 12, lines 258 to 260).

6. Page 13 The authors state “There was possible evidence of an increase in risk between years 8 and 14 after discharge, which may have reflected the underlying age of the veteran cohort.” Really? To my eyes that is not what Figure 1 shows. Year 14 is high but that is one year out of 16. Page 20 also mentions this.

**Response**: Thank you for your comment. On reflection, we agree with the reviewer and have removed that sentence from page 15 of the results section and have reworded page 21 of the discussion (lines 417 to 418):

“We found some year-on-year variation in the suicide rate in the years after discharge from the UKAF but the risk of suicide was fairly persistent.”

7. Page 18, Table 3 This seems to have some entries in bold. If you are going to do that then do it consistently across all the tables but I would suggest it is not a good idea. Leave it to the readers to decide what to look at.

**Response**: As suggested, we have removed bold text from the statistical values in Table 3.

8. I applaud the attempt to give the exact p–values but it does make the table rather ugly. More decimal places might help, there is room.

**Response**: As suggested, p-values up to 3 decimal points have been added to Table 3.

9. Page 18, Table 3 I do not understand the footnote about χ 2 . Are these not supposed to be from a conditional logistic regression? Are these really unadjusted for covariates?

**Response**: Thank you for your comment. The footnote in Table 3 has been removed for clarity. To confirm, the model ORs were unadjusted, but they were matched on age, gender and year of death.

10. Page 20 If suicide methods are sex dependent then does it make sense to compare general populations? I agree this may mean we can only see male results but they would be less contaminated.

**Response**: Thank you for your comment. Overall findings (males/females combined) on method of suicide have been provided due to the very small number of female veterans in our cohort. To provide male only figures would divulge the small number of different methods of suicide in the female group and we would be unable to do this due to the MoD disclosure controls we followed. However, the proportion of deaths by hanging, self-poisoning and firearms are the same in male veterans as they are in veterans overall – reflecting the much larger proportion of male veterans in our cohort.

11. Supplement I suspect RECORD (Benchimol et al., 2015) may be the more appropriate guideline. I did not notice any obvious omissions.

**Response**: Thank you for your comment. We did consider using the RECORD statement. However, the RECORD statement is specific to observational studies using routinely collected health data. The datasets used in our study are not routinely collected health data. The MoD Database is an administrative dataset of all service personnel who have left the UKAF and the NCISH database was created for a specific a priori research goal – not to routinely collect health data.

12. Age categories: Different analyses use different categorisations. I can see why that might be but an explicit statement might be needed. Some of the tables provide that.

**Response**: The reviewer is correct that different analyses used different age categories. The age-specific rate ratios in Tables 1 used 5-year age bands, except for the oldest age category (65-87) where numbers were small (n=6). The unadjusted HRs in Table 2 were also categorised into 5-year age bands, again except for the oldest age category (45+) where categories were collapsed due to the smaller number of suicide deaths in older veterans. In Table 4, age categories also used 5-year age bands, except for the oldest age category (45-54) where the number of in-service suicide deaths in male regulars aged over 45 years decreased to small numbers with increasing age. As the reviewer highlights, a statement on age categorisation was missing from Table 2 – this has now been added. An overall statement on age categorisation has also now been added to the statistical analysis section of the methods:

“In the oldest age-bands different age-categories were collapsed for some analysis because of small numbers” (see page 11, lines 235-236).

13. Age categories: Plotting with a superimposed curve (perhaps loess) might help enlighten us about the age relationships. Table 1 is an example. The plot could then use age in years which is less arbitrary than categorised age.

**Response**: Thank you for your suggestion. We have now added a Lowess smoothing curve to the main manuscript (see Figure 1). This shows that overall, the suicide rate in veterans appeared to fall with age. This has been added to the results section of the manuscript (page 14, lines 304-305). We have also added a statement about using a Lowess curve to the statistical analysis subsection of the methods (page 11, lines 236-238).

14. Models: Presumably if you are neither an officer nor an NCO you are a private soldier or equivalent. There seem to be a lot of them (Page 15, Table 2). Coupled with the high proportion discharged as untrained makes me suspect that many of these may have been early discharges. The length of service data seems to confirm that. Is that true? As a UK taxpayer I would hope our armed forces were not quite so untrained.

**Response**: Yes, the reviewer is correct. If an individual is neither a Commissioned Officer nor an NCO then they are of a non-officer rank (e.g. private soldier, able rate, marine, aircraftman). Individuals in a non-officer rank comprised 55% of our total cohort, commissioned officers 10% and NCOs 35%. We have now added a definition of “non-officer rank” as a footnote to Table 2. The reviewer is also correct that a significant proportion of the private soldiers who died (64%) served for less than 3 years – this is consistent with our existing messages about early service leavers.

15. Models: I think that:
a. The fact that these are unadjusted models needs specifying in the text not just in the caption.

**Response**: Thank you for highlighting this, we have now specified that unadjusted HRs were calculated from Cox proportional hazard models in the “statistical analysis” section of the methods on page 11, line 244.

b. The discussion needs to take account of the fact that, if I am correct, the correlated covariates are saying the same thing three times not three separate things.

**Response**: We agree that some of the covariates are correlated and there is significant overlap between factors such as younger age at discharge, short length of service, and being untrained. In some senses, the reviewer is correct that the findings are “saying the same thing”. We would frame this slightly differently and say that there is consistency in the findings, which seem to point to young people who serve for only a short period as being a vulnerable group. We have now added some text to this effect to the interpretation section of the discussion (page 24, lines 493-498).

c. If they are correlated the multivariable model could be fitted and the adjusted coefficients presented alongside the unadjusted ones which may prove enlightening.

**Response**: We are keen to avoid a multivariable model if possible. This was an a priori decision. We set out to explore specific associations between service-related factors and suicide. In the absence of specific hypotheses, we would be uncomfortable presenting a multivariable analysis. Inevitably such models are interpreted as showing that some risk factors are more important than others when that might not be the case.

16. It would also be better to provide an overall test for each covariate with more than two levels not just test levels against the reference.

**Response**: Thank you for your comment. Overall log-rank test results (p-values) are given for the covariates with more than two levels in the Cox regression models in Table 2. A footnote has now been included in Table 2, to clarify this.

**Reviewer #3:**

Thank you for inviting me to review this interesting paper and congratulations to the authors for producing a very interesting read, as well as additional pieces to the puzzle of suicide in servicemen and women.

1. ALL CAUSE MORTALITY AND KAPLAN-MEIER CURVES It would be very interesting to see data for all-cause mortality as well as Kaplan-Meier curves for suicide (within the UKAF population) stratified by the predictors.

**Response**: Thank you for your comment. We are unable to present all-cause mortality curves because we did not obtain data on veterans of the UKAF who died by other causes. This study was specifically focused on suicide mortality. We have already cited this as a limitation on page 23, lines 467-469. We have, however, now added Kaplan-Meier survival curves for the main covariates in the Cox regression models (age at exit, training, deployment on combat operations, and length of service) as a supplementary file to the manuscript (see S1_Figure) and added a statement on their use to the statistical analysis subsection of the methods (page 11, line 244-245). These curves are consistent with the HRs presented in Table 2.

2. DEPLOYMENT Isn't it highly likely that this is a double healthy worker effect, that the servicemen who are selected to be deployed go through additional physical and psychological testing before being deployed, and therefore cannot be expected to be comparable to either the general population or the non-deployed population (where rejects also are included)? This has been found in Scandinavian deployed veterans, who have lower suicide risk than age-sex-matched general population but similar suicide risk when matched to comparators of similar intelligence, psychological evaluation scores and other physical and mental attributes (Pethrus et al [Sweden] and Pethrus et al [Sweden, Norway, Denmark], 2022).

**Response**: We agree with the reviewer. Individuals with physical or psychological health problems will be less likely to be admitted to serve in the UKAF, and less likely to be deployed on combat operations once serving. We like the reviewer’s conceptualisation of this as a ‘double healthy worker’ effect and have added it to the interpretation of the lower risk in deployed individuals. We have also quoted the Pethrus et al studies (see page 25, lines 517-520).

3. YOUNG AGE AS RISK FACTOR It would be interesting to see more discussion about this group in terms of selection. Many of these young servicemen appear to have been discharged for medical reasons, discipline or admin reasons. May these early discharges be indicators of not well-functioning young men? While the protective effects seen among the servicemen >35y may be an indicator of well-functioning individuals, as evidenced by their ability to function for a longer period in their job?

**Response**: We agree further discussion of the young age group is warranted. The risk is reflected in several variables (for example, age, length of service, training) – see also our comments to reviewer 2 above. It seems plausible that our findings in this group partly reflect a selection effect – that is pre-service vulnerability rather than in-service or post discharge exposures. We have included additional text in our interpretation section (see page 24, lines 496-498).

4. MENTAL HEALTH COHORT: Comparison numbers for health care contacts by age? It is described that UKAF and GenPop had similar contact% with mental health services (25% and 27%). For the youngest age groups, I find only data for one group (I guess the UKAF group; 10% for 16-19y and 23% for 20-24y). It would be nice to see both UKAF and GenPop data for these age groups.

**Response**: As suggested by the reviewer, we have added the proportion of 16-19 year olds and 20-24 year olds in recent contact with mental health services in the general population to the results section of the manuscript on page 17, lines 372-373. For information, this was: 18% of 16-19 year olds and 23% of 20-24 year olds. We have also added some text to the results section to caution that the numbers in the youngest age categories were small, so these proportions should not be interpreted as suggesting young veterans were less likely to have contact with mental health services than young people in the general population (see page 17, lines 374-376).

5. CAUSAL LANGUAGE: Causal language is often used such as "increased the risk of suicide". It may be better to use non-causal language such as "was associated with higher/lower risk".

**Response**: Thank you for your comment. We have amended our use of language, as suggested, where appropriate throughout the manuscript.

**Reviewer #4:**

Overall, this is a welcome, well constructed paper which covers an important topic and uses high quality data. It is definitely worthy of publication with some minor amendments as below.

1. In the abstract it is unclear to me why it is not made clear that the SMR was lower for all veterans than the general population (as CIs do not include 100). This should be corrected.

**Response**: As requested, the “methods and findings” section of the abstract has been amended to read:

“The overall rate of suicide in veterans was slightly lower than the general population (SMR [95% CI] 94 [88-99]).”

2. In the financial disclosure section any of the authors are paid (salary) by the MOD should be disclosed (e.g. KH). The same should go for authors who are paid by other government departments (e.g. AB).

**Response**: Thank you for your comment. The funding section has now been removed from the main manuscript following editorial request #6. We will ensure the funding and conflicts of interest sections within the article metadata accurately reflect those authors whose salaries are paid by Government departments.

3. In the introduction, it would be helpful to mention the Contact Group and the RC Psych Quality Improvement for Veterans MH services as initiatives which also have aimed to improve the care available for veterans [given that this topic is discussed].

**Response**: We have added the following text to the introduction (page 6, lines 128-131) regarding the QNVMHS and Contact Group, as highlighted by the reviewer:

“In addition, the Quality Network for Veterans Mental Health Services (QNVMHS)[31], developed in collaboration with the Contact Group [32], aims to improve mental health services and care for veterans.”

4. In the study setting it would be helpful to explain that the one day post service was used as this is the accepted UK definition - otherwise readers who do not know this might be perplexed why this definition was used.

**Response**: Thank you for your comment. The following text has been added to line 159, page 8.

“There was no restriction on length of military service; we included anyone who left after their first day of basic training**,** in accordance with the UK definition of a veteran [33].”

Please also see our response to Reviewer #1, comment 11.

5. Why was data on deployment to Northern Ireland not included. Many of those who might have died by suicide could well have served in what was a very challenging operation. This should be corrected if at all possible. Also important, but perhaps less so, are the operations in the former Yugoslavia. Again, this was a major campaign and should be included.

**Response**: Thank you for your comment. The Service Leavers Database (SLD) extract that we obtained from the Ministry of Defence included all personnel who left the UKAF between 1996 and 2018 and included markers for Gulf 1, the Falklands Campaign, Iraq and Afghanistan. Data on whether a veteran had served in Northern Ireland or in the former Yugoslavia were not included in the data extract that we received. However, that is not to say some of the veterans in our cohort had not served in these campaigns – it is that there was no marker for us to identify them in the SLD dataset.

6. With the controls, might they have served as a reserve? If so, can reserves be excluded as comparators.

**Response**: To clarify, our controls were individuals who had died by suicide within 12 months of mental health service contact and had *no* record of serving in the UKAF. This is specified on lines 250-251 of the statistical analysis subsection of the methods.

7. In the results - Officers and NCOs should not be conflated as Officers. If needed saying NCO or Commissioned Officers or rank higher than Corporal or equivalent would be better.

**Response**: We welcome your suggestion and have amended the text on page 16, lines 344-346 to read:

“Being married, being a commissioned officer, being an NCO (non-commissioned officer) and deployment on combat operations were associated with lower suicide risk.”

We have also amended Table 2 to refer to “Commissioned Officer”.

8. In the discussion - again there have been improvements in the NHS care delivered but also matching improvements with charitable care which now has a QI (RC Psych) kitemark and a wider range of charities delivering NICE care (Contact has helped with this).

**Response**: Thank you for your comment regarding the Royal College of Psychiatrists quality mark for the delivery of veterans’ mental healthcare. The following text has now been added to page 26, lines 551-552 of the manuscript.

“The QNVMHS also provides accreditation to NHS and third sector mental health providers that meet agreed quality standards for military mental healthcare.”

9. The 4 years to seek help is at odds with a paper looking at Combat Stress attendees which found that it was 2 years to seek help (van Hoorn LA et al, 2013).

**Response**: Thank you for highlighting this. In looking again at the references, we note that one reference (Moller et al, 2020) relates to Danish veterans who received help (not sought help) after start of deployment (and not after discharge), with the majority (61%) receiving help more than 4 years after start of deployment. However, the other reference (House of Commons Defence Committee, 2019) suggests it takes an average of 4 years for veterans to seek help, although this seems to depend on where the veteran served, with veterans of the Afghanistan conflict taking two years to seek help after leaving service, and Iraq veterans three to four years. For clarity, we have therefore removed the Moller reference and replaced with the van Hoorn et al reference, and have amended the text (page 26, lines 564-565), to read:

“Evidence suggests it takes an average of two to four years for veterans to seek mental health support.“
