# Supplementary material for: Suicide after leaving the UK Armed Forces 1996–2018: A cohort study
Source: PLoS Med. 2023 Aug 8;20(8):e1004273. doi: 10.1371/journal.pmed.1004273 (PMC10409259; doi:10.1371/journal.pmed.1004273)
Supplement: Attachment — Submitted filename: Suicide study review R2.doc [file pmed.1004273.s005.doc]

Thank you for sending the revised version of this paper and the authors’ response to the points that I and the other reviewers raised. The paper has been substantially improved and I am content that the “take-away message” is the right one to be going forward to inform policy. The section on interpretation of the findings in particular is greatly improved, as is the discussion of the strengths and limitations, and the plans for future work.

I do still have some concerns as to whether like is being compared with like in the general population comparison (Table 1), as Ref 42 does not give an age breakdown of the population, and lines 476-477 only refer to an aggregated number of suicide deaths in the general population. Therefore it is difficult to see how comparable figures for PY at risk by age at death could have been derived for the general population – perhaps the authors could clarify this?

Another important issue which needs to be highlighted is the relatively short follow-up time of only a maximum of 23 years for those who left the Armed Forces at the start of the study period, and much less for those who left later. Although this is mentioned under Limitations, it would benefit from further elaboration of the consequences, specifically that with a median age at discharge of 26 years, many of the leavers would not have reached middle age by the end of the study. This may have lowered the apparent risk of suicide in the older age-groups, and should be commented on as a limitation.

It would also be worth mentioning explicitly that those who leave at older ages (especially over 40) are likely to be officers or senior NCOs, who have been shown in Table 2 to be at reduced risk. Therefore seniority/rank (and by implication, education and socioeconomic status) is likely to be a factor in explaining the lower risk of suicide seen in the older age groups.

A further point which is worthy of comment is the relatively small number of suicides per year in some of the age categories, eg less than two per year averaged over the 23-year period in four of the age categories in Table 1. This may have impacted on the statistical significance of some of the findings.

As a separate piece of work (although not for this paper) there was a peak in suicides in the under-20s in the serving population in the mid-1990s, as reported in the Defence suicide reports. It would be worth the authors looking at whether this cohort remained vulnerable on leaving the Armed Forces, and indeed conducting an age-period-cohort analysis for the dataset as a whole to see whether there have been specific vulnerable subgroups over time.

In specific points:

Abstract L37: It would be better to specify “information on health service contacts was only available for specialist NHS mental health services . . .”. As written, it could be interpreted as applying to the entire study cohort.

P9 L193: The comma after Y34 should be removed and “excluding Y33.9” should be put in parentheses, to clearly link the exclusion to the Y10-Y34 code group.

P11 L226 et seq. (Statistical analysis). This section should also specify that PY at risk was calculated from date of discharge to suicide, or the end of the study if no suicide. It is not clear whether people experiencing non-suicide death were censored if they died from other causes (eg disease), ie competing risks, or whether PY at risk was calculated to the end of the study for all non-suicides.

P13 L291. For clarity, the last sentence should be amended to read “ . . aged 24 years and younger at the time of suicide”.

P21 L434 (Discussion). It would be useful to add more detail after “More likely in cases than controls (3% vs 1%)”, ie “although confidence intervals were very wide, OR 3.29, 95% CI 1.37-7.87”, to further illustrate the uncertainty raised by the very small number of veterans (nine) with this diagnosis.

P24 L489 (Discussion). The article related to Reservists (Ref. 45) was specific to returning from deployment, rather than “transition to civilian life” in the Regular sense, since under non-deployed circumstances they already live in the civilian world and therefore do not face the same unfamiliarity with that environment as Regulars may do on transitioning.

P25 L516-520 (Discussion). The concept of a “double healthy worker” effect is novel. As the early reports of a healthy worker effect were based on reduced mortality associated with length of employment (eg McMichael et al (1984)), in order for this to be valid it would be necessary for those who had deployed to have a lower mortality than those who did not deploy, after adjusting for confounders. However, deployment to a war zone is inevitably likely to involve an increased risk of mortality; in 2010, hostile action was the largest single cause of death in the UK Armed Forces (95 deaths, vs 5 suicides). Therefore this section needs to be very sensitively reworded to avoid misinterpretation, especially by veterans themselves and their families. As a technical point, being selected for deployment does not routinely involve additional testing; all military personnel are allocated an employability grading based on their routinely assessed medical fitness. Only those with a restricted grading are subjected to additional review prior to deployment, although this is usually based on medical records rather than “testing”. On balance, this entire sentence would be best omitted.

P25 L522. Line 217 (in the Methods) says that marital status came from the Service Leavers Database. With a documented association between divorce and suicide (eg Kposowa (2003)), were the cases still married at the time of suicide, which may be many years after leaving service? If not/unknown, it is plausible that the beneficial effect of being married could be diminished.

P25 L522. “ . . .having completed training” would be better than “having received training”. Those categorised as “untrained” will have received some training, but left before completing the full training programme.
