# Supplementary material for: Suicide after leaving the UK Armed Forces 1996–2018: A cohort study
Source: PLoS Med. 2023 Aug 8;20(8):e1004273. doi: 10.1371/journal.pmed.1004273 (PMC10409259; doi:10.1371/journal.pmed.1004273)
Supplement: Attachment — Submitted filename: Response to 2nd round reviewer comments.docx [file pmed.1004273.s006.docx]

**Manuscript ID: PMEDICINE-D-22-04050R1**

**Manuscript title: Suicide after leaving the UK Armed Forces 1996-2018: a cohort study**

**Responses to second round of reviewers’ comments**

**Editorial requests**:

1. At line 20 (abstract), you might wish to add a few words to explain the interest in exploring the research question now, given recent military engagements.

**Response: As suggested, the following text has been added to lines 20-21 of the abstract:**

**“This is important because the wider context of being a UK Armed Forces (UKAF) veteran has changed in recent years following a period of intensive operations”.**

2. At line 24, please add a sentence, say, to summarize the statistical methodology used.

**Response: As requested, a sentence summarising the statistical methodology has been added to lines 25-28 of the abstract, as follows:**

**“Retrospective cohort study of suicide in personnel who left the regular UKAF between 1996 and 2018 linking national databases of discharged personnel and suicide deaths, using survival analysis to examine the risk of suicide in veterans compared to the general population and conditional logistic regression to investigate factors most strongly associated with suicide after discharge.”**

3. Around line 24, we suggest quoting some additional information, such as the number of person-years at risk, the breakdown of the cohort by sex and age and the median length of follow-up.

**Response: As suggested, at lines 28-30 of the abstract the following text has been added:**

**“The 458,058 individuals who left the UKAF accumulated over 5,852,100 person years at risk, with a median length of follow-up of 13 years, were mostly male (91%), and had a median age of 26 years at discharge.“**

4. At line 41, please soften the wording by adapting the text to "In this study, we found that suicide risk in personnel leaving the UK armed forces was not high ..." or similar.

**Response: The wording has been amended, as requested.**

5. At line 42 and any other instances, please adapt the text to "... were associated with elevated risk ..." or similar.

**Response: Thank you for your comment. The wording has been amended, as requested.**

6. At line 43 and any other instances we suggest minor rewording so that "We/Our" is reserved for the authors, the text here becoming, e.g., "The focus ...".

**Response: Thank you for your comment. The wording has been amended, as requested. The wording has also been amended at line 589.**

7. We suggest using the active voice in a few elements of the Author summary, and suggest "We linked ..." at line 57.

**Response: The Author summary has been amended from “the researchers” to “we” at lines 56, 63 and 85.**

8. At line 151, please correct "STOBE".

**Response: Typo has been corrected, as requested.**

9. At line 300 you quote a raised suicide risk "two to three times higher" in younger groups. Is there an issue of consistency with findings quoted in the abstract here? Please correct if so.

**Response: Thank you for highlighting this inconsistency. Line 300 (“two to three times higher”) is correct. Findings quoted on lines 32 (abstract), 70 (author summary), and 423 (discussion) have been amended.**

10. Noting the word "gender" at line 426, and any other instances, would "sex" be more appropriate?

**Response: Thank you for highlighting this. We agree that “sex” would be more appropriate in this instance and line 434 has been changed to reflect this. We have also checked throughout the manuscript and have changed the wording from “gender” to “sex”, where appropriate.**

11. At line 575, we suggest "... and this is roughly equivalent ...".

**Response: Amendment has been made, as requested.**

**Reviewer #1:**

Thank you for sending the revised version of this paper and the authors’ response to the points that I and the other reviewers raised. The paper has been substantially improved and I am content that the “take-away message” is the right one to be going forward to inform policy. The section on interpretation of the findings in particular is greatly improved, as is the discussion of the strengths and limitations, and the plans for future work.

**Response: Thank you for your comments. We are pleased that you agree our manuscript has been improved following the insightful comments from reviewers.**

1. I do still have some concerns as to whether like is being compared with like in the general population comparison (Table 1), as Ref 42 does not give an age breakdown of the population, and lines 476-477 only refer to an aggregated number of suicide deaths in the general population. Therefore it is difficult to see how comparable figures for PY at risk by age at death could have been derived for the general population – perhaps the authors could clarify this?

**Response: Thank you for this comment – the reviewer is absolutely correct. Since this was not a case control analysis, like was not being compared with like. But that was not our aim (see pages 6-7, lines 145-148). We wanted to address the question of whether suicide rates in veterans were higher than the general population as a whole.**

**The reviewer is also right in highlighting the apparent inconsistency between the veterans’ suicide rates (calculated with a person-years denominator) with a general population rate (based on aggregated annual mid-year population estimates). However, this is an accepted and fairly standard approach to calculating SMRs (see Checkoway et al., 2004). An annual rate per 100,000 people based on a mid-year population estimate would be comparable to an annual rate expressed per 100,000 person years, assuming that each person in the general population contributes one-person year of follow up in that year (a reasonable assumption given the relative stability of the UK population). It would not have been feasible to calculate the actual person years at risk in the general population and it would not have changed our findings in any meaningful way.**

**We have amended the discussion to make the potential limitations of our approach absolutely clear (please see page 22, lines 464-469 and page 24, lines 498-501).**

2. Another important issue which needs to be highlighted is the relatively short follow-up time of only a maximum of 23 years for those who left the Armed Forces at the start of the study period, and much less for those who left later. Although this is mentioned under Limitations, it would benefit from further elaboration of the consequences, specifically that with a median age at discharge of 26 years, many of the leavers would not have reached middle age by the end of the study. This may have lowered the apparent risk of suicide in the older age-groups and should be commented on as a limitation.

**Response: Thank you for your comment. We agree that the age at discharge and the length of follow up warrants a specific mention and further elaboration of the potential consequences. Thinking about it some more though, we are not sure that this would lead inevitably to a lowering of the apparent suicide risk since as well as fewer deaths there would be fewer years of follow up for older age groups. What we can say is that this might make our estimates of risk in older age groups less precise (see also comment on oldest age groups below). This has been added as a limitation of the study on page 23, lines 491-496, as follows:**

**“Furthermore, veterans were followed-up for a maximum of 23 years for those who left the UKAF at the start of the study period (1996), less for those who left later. With a median age at last discharge from the UKAF of 26 years, many of the veterans we examined would not have reached middle-age by the end of the study period and those who did may not have been representative of all veterans in this age group. This means that our estimates of risk for older age-groups may be less precise than our estimates for younger age groups.”**

3. It would also be worth mentioning explicitly that those who leave at older ages (especially over 40) are likely to be officers or senior NCOs, who have been shown in Table 2 to be at reduced risk. Therefore seniority/rank (and by implication, education and socioeconomic status) is likely to be a factor in explaining the lower risk of suicide seen in the older age groups.

**Response: Thank you for your comment. We have added the following to page 25, lines 539-542 of the manuscript, as suggested:**

**“….although it is possible that older veterans were more likely to be officers or NCOs (higher rank was also protective) and rank (and by implication education and socioeconomic status) may be a factor in explaining the lower risk of suicide found in older veterans.”**

4. A further point which is worthy of comment is the relatively small number of suicides per year in some of the age categories, eg less than two per year averaged over the 23-year period in four of the age categories in Table 1. This may have impacted on the statistical significance of some of the findings.

**Response: As suggested by the reviewer, the following has been added to the strengths and limitations section of the manuscript (page 23, lines 488-491):**

**“In the oldest age categories (55-87 years) particularly there were fewer than an average of 2 suicide deaths per year over the study period and this may have impacted on the power of our study and the statistical significance of some of our findings.”**

5. As a separate piece of work (although not for this paper) there was a peak in suicides in the under-20s in the serving population in the mid-1990s, as reported in the Defence suicide reports. It would be worth the authors looking at whether this cohort remained vulnerable on leaving the Armed Forces, and indeed conducting an age-period-cohort analysis for the dataset as a whole to see whether there have been specific vulnerable subgroups over time.

**Response: This is an excellent and worthwhile study proposed by the reviewer which we will certainly explore. One issue may be statistical power. Resources permitting, we plan to investigate age- and sex-specific rates of suicide in veterans over time in future work.**

6. Abstract L37: It would be better to specify “information on health service contacts was only available for specialist NHS mental health services . . .”. As written, it could be interpreted as applying to the entire study cohort.

**Response: Thank you for highlighting this. The text on lines 42-43 has been amended to “…information on contact with support services was only available for veterans in contact with specialist NHS mental health services….”, as recommended by the reviewer.**

7. P9 L193: The comma after Y34 should be removed and “excluding Y33.9” should be put in parentheses, to clearly link the exclusion to the Y10-Y34 code group.

**Response: Thank you for your comment. This amendment has been made, as suggested.**

8. P11 L226 et seq. (Statistical analysis). This section should also specify that PY at risk was calculated from date of discharge to suicide, or the end of the study if no suicide. It is not clear whether people experiencing non-suicide death were censored if they died from other causes (eg disease), ie competing risks, or whether PY at risk was calculated to the end of the study for all non-suicides.

**Response: As the reviewer has suggested, we have now clarified (on page 11, lines 240-242) that person-years at risk was calculated from date of discharge to suicide or date of discharge to the end of study for those veterans that did not die by suicide:**

**“Person-years at risk for each veteran was calculated from the date of being discharged from the UKAF to the date of suicide, or to the end of the study period (31 December 2018), if the individual did not die by suicide.”**

9. P13 L291. For clarity, the last sentence should be amended to read “ . . aged 24 years and younger at the time of suicide”.

**Response: Thank you for your comment. This amendment has been made, as suggested.**

10. P21 L434 (Discussion). It would be useful to add more detail after “More likely in cases than controls (3% vs 1%)”, ie “although confidence intervals were very wide, OR 3.29, 95% CI 1.37-7.87”, to further illustrate the uncertainty raised by the very small number of veterans (nine) with this diagnosis.

**Response: As the reviewer has helpfully suggested, lines 442-444 have been amended to read:**

**“Evidence of a PTSD diagnosis was uncommon but more likely in cases than controls (3% vs 1%), although confidence intervals were wide (OR 3.29 (1.37-7.87)) given the small number of veterans (nine) with this diagnosis.”**

11. P24 L489 (Discussion). The article related to Reservists (Ref. 45) was specific to returning from deployment, rather than “transition to civilian life” in the Regular sense, since under non-deployed circumstances they already live in the civilian world and therefore do not face the same unfamiliarity with that environment as Regulars may do on transitioning.

**Response: Thank you for highlighting our error. The sentence on page 24, lines 514-516 has been amended to read:**

**“…although there is some evidence that deployed reservists may have poorer mental health outcomes than deployed regulars upon returning home from combat operations.”**

12. P25 L516-520 (Discussion). The concept of a “double healthy worker” effect is novel. As the early reports of a healthy worker effect were based on reduced mortality associated with length of employment (eg McMichael et al (1984)), in order for this to be valid it would be necessary for those who had deployed to have a lower mortality than those who did not deploy, after adjusting for confounders. However, deployment to a war zone is inevitably likely to involve an increased risk of mortality; in 2010, hostile action was the largest single cause of death in the UK Armed Forces (95 deaths, vs 5 suicides). Therefore this section needs to be very sensitively reworded to avoid misinterpretation, especially by veterans themselves and their families. As a technical point, being selected for deployment does not routinely involve additional testing; all military personnel are allocated an employability grading based on their routinely assessed medical fitness. Only those with a restricted grading are subjected to additional review prior to deployment, although this is usually based on medical records rather than “testing”. On balance, this entire sentence would be best omitted.

**Response: We agree with the Reviewer’s concerns about the potential sensitivity of the ‘double healthy worker’ effect. However, we have taken further advice from colleagues and have decided to retain this sentence, albeit it with significant rewording. In retrospect, we agree use of the word ‘testing’ was incorrect. The reviewer’s comments also highlighted to us that we needed to ensure it was clear that we were referring to suicide mortality not overall mortality. The wording is now as follows (see pages 25-26, lines 546-553):**

**“This finding could partly reflect what might be conceptualised as a “double healthy worker effect” with respect to suicide mortality – individuals with physical or psychological health problems will be less likely to be accepted into military service and may be less likely to be deployed on combat operations once in service. Although being selected for deployment does not routinely involve additional testing, postings selected for, or role within units, it could mean that more vulnerable individuals are less likely to be deployed or may already have left the UKAF. If this was the case, then those eligible for deployment would be healthier than those not deployed and healthier than those in the general population [14, 46]”.**

13. P25 L522. Line 217 (in the Methods) says that marital status came from the Service Leavers Database. With a documented association between divorce and suicide (eg Kposowa (2003)), were the cases still married at the time of suicide, which may be many years after leaving service? If not/unknown, it is plausible that the beneficial effect of being married could be diminished.

**Response: Thank you for your comment. The reviewer is correct that marital status came from the Service Leavers Database. It is therefore plausible that marital status may have changed at the time of suicide, especially if the suicide death occurred many years after leaving service. We have acknowledged this on page 26, lines 555-559 of the manuscript as follows:**

**“Other protective factors included being married and having completed training, perhaps highlighting the importance of social support and integration. However, we acknowledge the beneficial effect of being married could be diminished in those individuals whose marital status changed between discharge and death (as information on marital status was obtained from MOD administrative systems related to time in service).”**

14. P25 L522. “ . . .having completed training” would be better than “having received training”. Those categorised as “untrained” will have received some training, but left before completing the full training programme.

**Response: Thank you for your comment. This amendment has been made, as suggested.**

**Reviewer #3:**

Thank you for a thorough revision and well-motivated answers to the reviewer comments. I only have minor comments at this stage and look forward to hopefully seeing this interesting paper out in the public domain soon.

1. ABSTRACT CONCLUSION: "Conclusions Suicide risk in veterans is not high but there are important differences according to age, with higher risk in young men and women. We found a number of factors which increased the risk of suicide but deployment was associated with reduced risk."

COMMENT: Causal language is still used ("increased the risk" and "reduced risk"). I would suggest using HIGHER instead of "increased" and LOWER instead of "reduced".

**Response: Thank you for your comment. The sentence has now been amended to read:**

**“We found a number of factors which elevated the risk of suicide but deployment was associated with lower risk." Please also see editorial comment #5.**

2. DISCUSSION, MAIN FINDINGS: "Methods of suicide in veterans were comparable to the general population, although hanging and strangulation were more common (62% versus 44%) and self-poisoning less common (14% versus 24%) in veterans - potentially reflecting the gender composition of the veteran cohort compared with the general population."

Are there not GenPop statistics split by sex so that a comparison of male veterans with men in the general population can be done?

**Response: Thank you for your comment. The reviewer is correct that general population suicide statistics (such as those published by ONS) provide information on method of suicide by sex. However, we have provided overall findings (males/females combined) on method of suicide due to the very small number of female veterans in our cohort. If we provided male-only figures this would divulge the small number of different suicide methods in female veterans. We would be unable to do this due to the MOD disclosure controls that we followed.**

3. STRENGTHS AND LIMITATIONS: "... may continue to improve health outcomes and reduce suicide risk in this vulnerable population."

QUESTION: What vulnerable population is referred to - is it the young ones who were discharged early? Because overall the veterans do not seem to be a vulnerable population, judging from the lower SMR.

**Response: Thank you for your comment. The text on line 608 has now been amended to**	**read:**

**“…may continue to improve health outcomes and reduce suicide risk in, particularly younger, veterans.”**
